# Transcriptomic Analysis Revealed Candidate Genes Involved in Pseudomale Sperm Abnormalities in Chinese Tongue Sole (*Cynoglossus semilaevis*)

**DOI:** 10.3390/biology11121716

**Published:** 2022-11-26

**Authors:** Yuxuan Sun, Ming Li, Zhongkai Cui, Mengqian Zhang, Tingting Zhang, Lu Li, Na Wang, Xiwen Xu, Min Wei, Wenteng Xu

**Affiliations:** 1 Yellow Sea Fisheries Research Institute, Chinese Academy of Fishery Sciences (CAFS), Laboratory for Marine Fisheries Science and Food Production Processes, Pilot National Laboratory for Marine Science and Technology, Qingdao 266071, China; 2 School of Marine Science and Fisheries, Jiangsu Ocean University, Lianyungang 222005, China

**Keywords:** *Cynoglossus semilaevis*, spermatogenesis, transcriptomic profile, FoxO signalling pathway, sex chromosome biased distribution

## Abstract

**Simple Summary:**

Chinese tongue sole (*Cynoglossus semilaevis*) is an important aquatic fish in northeast Asia that displays sexual growth morphism, with females being 2–4 times larger than males. Tongue sole has a ZZ/ZW sex determination system, and the genotypic female (ZW) can be sex-reversed into phenotypic males, namely, pseudomales. Pseudomales show similar growth to male fish and are disadvantageous in aquaculture. Moreover, pseudomale fish can produce sperm but only Z-type sperm, and its epigenetic information makes its ZW offspring prone to become pseudomale. Thus, screening the key genes for W absence would provide clues for producing high female ratio or all female fry, benefiting the tongue sole industry. In this study, we compared the transcriptomic profiles of pseudomale and male sperm and found that the FoxO signalling pathway, especially the *foxo3a* and *foxo6-like* genes, may play important roles in spermatogenesis. In addition, we identified an ~1 M bp area on the Z chromosome enriched with eight DEGs. The study has provided valuable data for screening candidate genes involved in pseudomale sperm abnormity, and their functional study in the future would shed light on sex control in the tongue sole industry.

**Abstract:**

Chinese tongue sole (*Cynoglossus semilaevis*) has a ZZ/ZW sex determination system, but the genotypic female (ZW) can be sex-reversed into phenotypic males, namely, pseudomales. Pseudomale fish can produce only Z-type sperm but not W sperm. However, the molecular mechanism is unclear. To screen the key genes involved in pseudomale sperm abnormalities, we analysed the transcriptomic profiles of pseudomale and male sperm. In comparison to male sperm, 592 differentially expressed genes (DEGs) were identified in pseudomale sperm, including 499 upregulated and 93 downregulated genes. KEGG analysis indicated that the FoxO signalling pathway, especially the *foxo3a* and *foxo6-like* genes, may play an important role in spermatogenesis. The DEGs were mainly distributed on sex chromosomes, with 158 downregulated genes on the Z chromosome and 41 upregulated genes on the W chromosome. A specific area (14–15 M) on the Z chromosome was identified, which enriched eight DEGs inside the ~1 M region. In addition, there were five gene alleles on the sex chromosomes, which showed the opposite transcription pattern (upregulated for the W allele, downregulated for the Z allele). This study has provided valuable data for screening candidate genes involved in the pseudomale sperm abnormality.

## 1. Introduction

Chinese tongue sole (*Cynoglossus semilaevis*) belongs to order Pleuronectiformes, family Cynoglossidae. Due to its delicious taste, it is now an economically important marine flatfish in Northeast Asia, such as China, South Korea and Japan. Chinese tongue sole exhibits dramatic sex dimorphism, where female individuals (ZW type) can grow 2–4 times larger than males (ZZ type) [1], so a high-female-ratio or all-female fry would benefit tongue sole aquaculture. However, under special environmental conditions (e.g., high temperature), genotypic females of Chinese tongue sole can be sex-reversed into phenotypic males, namely, pseudomales [2,3]. Pseudomales display slow growth similar to that of male fish, which is disadvantageous for aquaculture. In theory, pseudomale fish should produce both Z and W sperm, and crossing them with females would increase the female ratio from 50% to 75% (ZZ:ZW:WW = 1:2:1). However, pseudomale sperm contains only Z-type, and W sperm are lacking. In addition, the pseudomale sperm inherit parental epigenetic information, which results in the ZW offspring of pseudomales being prone to becoming pseudomales [4].

In humans, over 2000 genes are involved in spermatogenesis, and defects in these genes may lead to sperm abnormalities or male infertility [5]. However, the lack of a specific type of sperm (W sperm absence) is a unique phenomenon in vertebrates. Thus, screening key genes and dissecting their regulatory network during spermatogenesis would provide clues for the W sperm absence, which has great application potential for producing high-female-ratio or all-female fry.

In Chinese tongue sole, there are many genes reported to be involved in spermatogenesis, especially those localized on the Z chromosome, such as dmrt1, tesk1, and neurl3 [3,6,7]. Large-scale analysis was performed to study the molecular mechanism of sex reversal [8,9,10]. However, most research attention is focused on gonadal differentiation, and a comparison of transcriptomic profiles between pseudomale and male sperm is still lacking. In this study, we conducted transcriptomic analysis of pseudomale and male sperm, and the differentially expressed genes displayed a special distribution pattern.

## 2. Materials and Methods

### 2.1. Sample Preparation

Chinese tongue sole was purchased from Haiyang High-Tech Experimental Base (Haiyang, Shandong Province, China). Sperm was obtained from male and pseudomale fish (1.5 years old, each sex included four individuals; average body weight and length for male 176.2 g, 30.8 cm, and for pseudomale, 177.4 g, 30.9 cm); it was transferred to liquid nitrogen immediately and then stored at −80 °C until RNA isolation. Regarding the sample size, we actually obtained 13 pseudomale individuals. However, pseudomale produced less sperm, and at last, only four samples were qualified for both quality and quantity. Despite this, the transcriptomic data showed high consistency, and qPCR results showed their reliability, so we conducted the analysis by using four males and four pseudomales. Fins were also collected from the tail for genomic DNA extraction. All experimental procedures were approved by the Yellow Sea Fisheries Research Institute’s animal care and use committee.

### 2.2. Genetic Sex Identification

Genomic DNA was extracted from the fins and stored in absolute ethanol using a TIANamp Marine Animals DNA kit (Tiangen, Beijing, China) following the manufacturer’s instructions. Genetic sex identification was performed using specific primers as previously described [11]. In brief, specific primers were designed for PCR (Sex F: CCTAAATGATGGATGTAGATTCTGTC; Sex R: GATCCAGAGAAAATAAACCCAGG), and the resultant product was examined by 4% agarose gel electrophoresis. For female samples, two bands (169 and 134 bp) could be visualized, while only one band (169 bp) could be observed for male samples.

### 2.3. Total RNA Extraction and Library Preparation

Total RNA was extracted using TRIzol (Invitrogen, Carlsbad, CA, USA) reagent based on the manufacturer’s protocol. RNA purity and quantification were evaluated using a NanoDrop 2000 spectrophotometer (Thermo Scientific, Waltham, MA, USA). Within each group, four males and four pseudomales were used for RNA isolation. Thus, eight cDNA libraries of M1-4 and PM1-4 were constructed.

### 2.4. RNA Sequencing and Differentially Expressed Gene (DEG) Analysis

Transcriptome sequencing and analysis were conducted using the Illumina platform by OE Biotech Co., Ltd. (OE Biotech, Shanghai, China), and the raw data (raw reads) were submitted to NCBI (Accession: PRJNA894095). Raw data were processed using Trimmomatic (0.36) [12]. The reads containing poly-N and the low-quality reads were removed to obtain clean reads. Then, the clean reads were mapped to the reference genome using hisat2 [2,13] under the default settings except ‘--rna-strandness rf --fr’. The fragments per kilobase million (FPKM) value of each gene was calculated using Cufflinks [14] and the read counts of each gene were obtained by htseq-count (0.9.1) [15] with the parameter ‘-s reverse’. Differential expression was analyzed using the DESeq (1.18.0) R package functions estimateSizeFactors and nbinomTest (http://www.bioconductor.org/packages/release/bioc/html/DESeq.html (accessed on 16 November 2022)). Genes with fold change >2 or fold change <0.5 and *p* < 0.05 were considered DEGs. The DEGs were then enriched by Gene Ontology (GO) terms and Kyoto Encyclopedia of Genes and Genomes (KEGG) categories using the clusterProfiler (4.6.0) R package based on the hypergeometric distribution (https://www.bioconductor.org/packages/release/bioc/html/clusterProfiler.html (accessed on 16 November 2022). Transcriptomic data of testis pseudomales and males were obtained from a previous study by our group [16].

### 2.5. qPCR Validation

To validate the transcriptomic data, seven genes were randomly selected (the primers are shown in Supplementary Table S1), and qPCR was performed with a 7500 Fast machine (Applied Biosystems, Foster City, CA, USA) using TB Green Premix Ex Taq (Tli RNaseH Plus) (Takara, Japan) according to the manufacturer’s’ protocols. *Β-actin* was used as an internal reference for normalization. Each individual had four repetitions, and the relative transcription level was calculated by the 2^−ΔΔCt^ method. All data are presented as the mean ± SD, and differences between the means were tested by analysis of variance (ANOVA) followed by Duncan’s post hoc test using SPSS software. Significant differences were accepted when the *p* value ≤ 0.05. In addition, qPCR was also performed to analyze the transcriptomic profile of five *foxo* genes (the primers are shown in Supplementary Table S1).

## 3. Results

### 3.1. Transcriptomic Overview and Identification of DEGs

**Figure 1:**
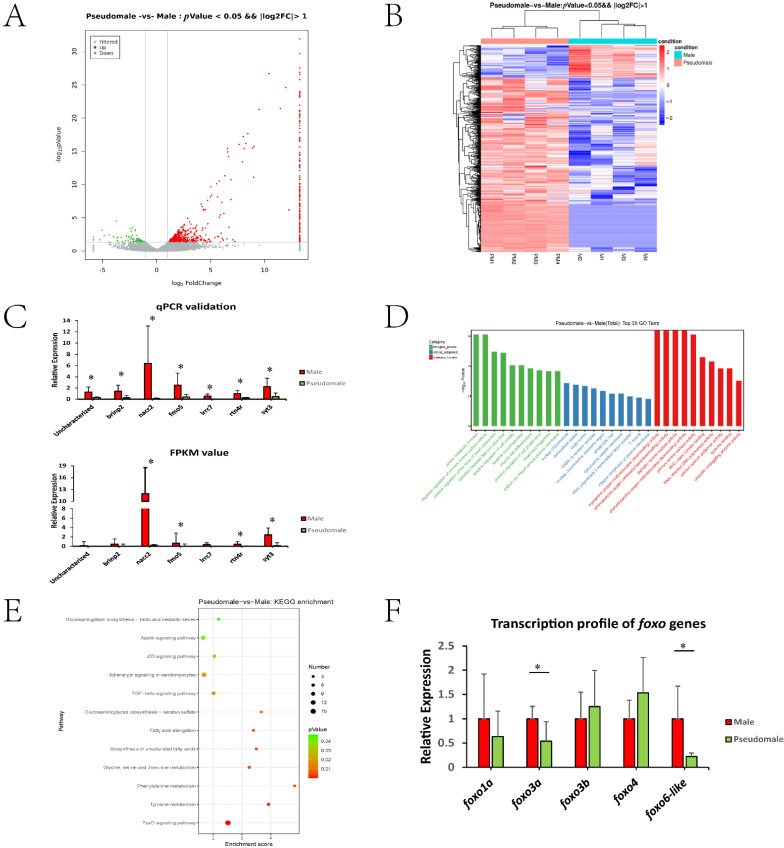
(**A**) Volcano plot of DEGs. (**B**) Heat map of DEGs. (**C**) qPCR and transcriptomic profile of seven randomly selected genes between male and pseudomale sperm. GO (**D**) and KEGG analysis (**E**) of DEGs between the pseudomale and male sperm transcriptomes. (**F**) The transcription profile of five *foxo* genes between the male and pseudomale sperm. * indicated significant differences (*p* < 0.05).

As shown in Supplementary Table S2, eight sperm samples (four male and four pseudomale individuals) were used, producing raw data ranging from 6.43 to 6.90 Gb. The average rate of valid bases was 96.70%, and the clean reads of Q30 were all above 92%. As shown in Supplementary Table S3, we obtained 592 DEGs, including 499 upregulated and 93 downregulated genes (pseudomale versus male). From the volcano plot, we found that most DEGs showed fold change ranging from 2–8 (Figure 1A). In Figure 1B, heatmap indicated the repeatability among samples and those genes showing high expression in male but low expression in pseudomale should be focused upon. The qPCR data exhibited a profile consistent with the FPKM value, indicating the reliability of the transcriptomic data (Figure 1C).

### 3.2. GO and KEGG Enrichment

As shown in Figure 1D, the top 30 GO terms were listed based on three categories: biological process, cellular component, and molecular function. The GO terms were mainly involved in basic metabolic processes, such as amine metabolism and different amino acid deaminating processes. However, several terms (predominantly in the cellular component category, such as nuclear chromosome, extracellular space, Golgi lumen and so on) were also enriched, which might be related to the formation or maintenance of the sperm structure. The KEGG enrichment data are shown in Figure 1E. The pathways could be mainly categorized into three groups: fatty acid metabolism (Biosynthesis of unsaturated fatty acids, Fatty acid elongation, Apelin signaling pathway, Glycosphingolipid biosynthesis), amino acid metabolism (Tyrosine metabolism, Phenylalanine metabolism, Glycine, serine and threonine metabolism), and other pathways (Glycosaminoglycan biosynthesis, TGF-beta signaling pathway, Adrenergic signaling, p53 signaling pathway, FoxO signaling pathway). It is worth noting that the FoxO signalling pathway exhibited significant enrichment. Based on these observations, the tongue sole genome was screened, and five *foxo* genes were identified: *foxo1a*, *foxo3a*, *foxo3b*, *foxo4*, and *foxo6-like*. Their transcription profiles were examined by qPCR, and *foxo3a* and *foxo6-like* showed a significant decline in pseudomale sperm, suggesting their role in pseudomale sperm abnormalities (Figure 1F).

### 3.3. Chromosomal Distribution Pattern of DEGs

**Figure 2:**
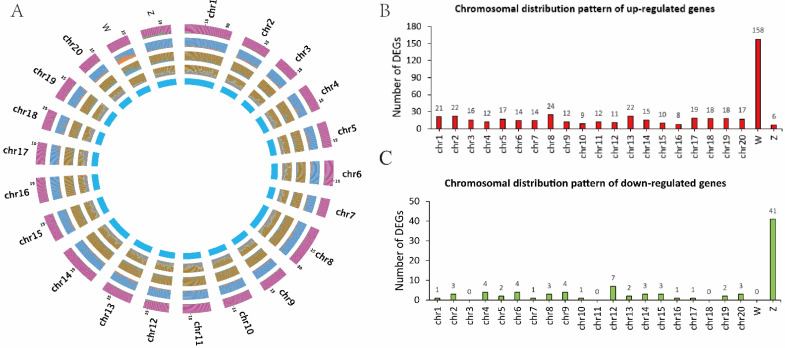
The chromosomal distribution of DEGs from sperm transcriptomes. (**A**) The circus plot of the genome features. From outer to inner: A distribution of downregulated genes; B, distribution of upregulated genes; C, distribution of DEGs; D, gene density; E, representing the 24 chromosomes. (**B**) The number of upregulated DEGs on each chromosome. (**C**) The number of downregulated DEGs on each chromosome.

**Figure 3:**
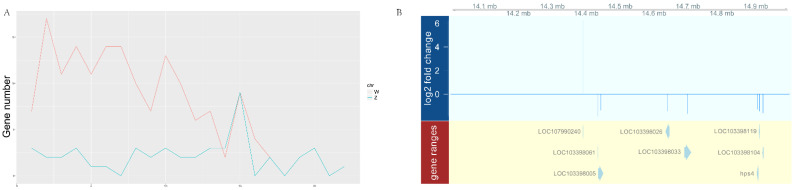
The distribution pattern of sperm DEGs on the Z chromosome. (**A**) The number of DEGs every 1 M. (**B**) Genes localized in the 14–15 M region of the Z chromosome.

The chromosomal distribution of DEGs was further analyzed. As shown in Figure 2, the number of DEGs showed no big fluctuation among autosomes, while a biased DEG distribution on the sex chromosomes was observed. Among the 499 upregulated genes, 158 were localized on the W chromosome. Of the 93 downregulated genes, 41 were localized on the Z chromosome. Moreover, we found that the DEGs were not evenly distributed on the sex chromosomes and instead were specifically enriched in the 14–15 M region of the Z chromosome (Figure 3A). As shown in Figure 3B, eight genes were mapped in this region, including one upregulated and seven downregulated genes. In fact, there were also eight genes upregulated in the corresponding region of the W chromosome. The accumulation of DEGs in this “hot area” might be the result of epigenetic regulation, as pseudomale testis showed hypermethylation in this area compared to male (Supplementary Figure S3 and unpublished data). Interestingly, in pseudomales, we also identified five gene alleles on the sex chromosomes displaying opposite expression patterns, that is, upregulation for the W allele but downregulation for the Z allele (Table 1).

## 4. Discussion

Transcriptome analysis has been widely used as an exploratory experimental method to reveal key genes of biological traits. Corresponding software, such as Hisat2 (for reads mapping) and DESeq (for DEGs analysis), has been used to provide new insights into the mechanism of disease resistance, stress resistance, sex determination and differentiation or sex reversal of tongue sole [9,17,18,19].

As an important marine fish in northeast Asia, the tongue sole industry is limited by its female ratio in the aquatic population. Gynogenesis is the most frequently used method in sex control. Here we use tongue sole as example; gynogenesis should produce ZZ and WW fry. However, WW fry is unable to survive and develop [1]. Thus, pseudomale could be an alternative for breeding. In theory, pseudomale crossed with female should produce three genotypes (ZZ:ZW:WW = 1:2:1). Although WW fry cannot survive, the female ratio should reach 66.7% (ZZ:ZW = 1:2). Unfortunately, we have found there was no W sperm in pseudomales, so uncovering the mechanism of W sperm absence has both scientific significance and applicable potential. Azoospermia is frequently reported in mammals, while a lack of specific sperm, such as pseudomale tongue sole producing Z-type sperm but no W-type sperm, is an uncommon phenomenon. To elucidate the mechanism of W sperm absence, studies have focused on the gonadal level, not on sperm level. Since the pseudomale could produce sperm, we compared the transcriptomic profile between pseudomale and male sperm. The W chromosome only existed in the pseudomale, so the transcription level of W-linked genes was not comparable between males and pseudomales. For this reason, we predominantly focused on DEGs of the Z chromosome. In pseudomale sperm, most of the downregulated genes were enriched on the Z chromosome. A similar pattern was also observed in pseudomale testis, with 75 of 132 downregulated genes on the Z chromosome (Supplementary Figure S1). According to our previous postulation, many Z chromosome genes are involved in male determination/differentiation and spermatogenesis, so their downregulation might account for abnormal pseudomale spermatogenesis.

Furthermore, we identified a specific region on the Z chromosome, and eight DEGs were enriched within an approximately 1 M area (Figure 3 and Supplementary Table S4). However, these genes were mainly involved in energy metabolism and transcription based on their annotation, and their functionality in spermatogenesis requires further investigation. Interestingly, another eight DEGs were also identified to be enriched in this same area of the Z chromosome in pseudomale testis, but they were totally different from these DEGs from pseudomale sperm compared to male testis (Supplementary Figure S2 and Supplementary Table S5), so this region might accumulate genes accounting for gonadal differentiation and spermatogenesis. Furthermore, we identified five gene alleles showing the opposite expression pattern: upregulation on the W allele but downregulation on the Z allele (Table 1). To our knowledge, only the transcriptional activator protein Pur-beta-like (LOC103396891) has been suggested to be associated with sexual dimorphism (data not shown), while its role in spermatogenesis is unclear. Therefore, functional study of these genes during spermatogenesis is still needed.

It is interesting that the FoxO signalling pathway was identified via KEGG analysis. There have been a number of reports about the participation of the FoxO signalling pathway and *foxo* genes in spermatogenesis in mammals. Among them, *foxo1* is conserved in mammalian species and has been suggested to play an important role in spermatogonial stem differentiation and spermatogenesis [20,21,22,23,24,25]. We also analyzed the expression patterns of the five *foxo* genes in early gonadal stages, all of which showed male-biased expression (data not shown). Thus, we postulate that the FoxO signalling pathway might not only function in spermatogenesis but also participate in early testicular differentiation (Figure 1C,D, and Supplementary Figure S4).

## 5. Conclusions

Comparative transcriptomic analysis of tongue sole sperm identified 592 DEGs (pseudomale versus male, 499 up and 93 down). The DEGs were predominantly enriched on sex chromosomes, with 158 downregulated genes on the Z chromosome and 41 upregulated genes on the W chromosome. A specific ~1 M region (14–15 M) on the Z chromosome harbored eight DEGs. Moreover, the FoxO signalling pathway plays an important role in spermatogenesis, and the functions of *foxo3a* and *foxo6-like* should be considered.

## Figures and Tables

**Table 1:** Information on five gene alleles on the sex chromosomes.

Gene ID	Localization	Annotation	Gene ID	Localization	Annotation
LOC103397162	W	ankyrin repeat domain-containing protein 13A-like	LOC103397320	Z	ankyrin repeat domain 13A
LOC103397197	W	SET-binding protein-like	LOC103398277	Z	SET binding protein 1
LOC103397030	W	ras-related protein Rab-35-like	LOC103397428	Z	ras-related protein Rab-35-like
LOC103396891	W	transcriptional activator protein Pur-beta-like	LOC103397871	Z	transcriptional activator protein Pur-beta
LOC103396909	W	G protein-coupled receptor kinase 5-like	LOC103398033	Z	G protein-coupled receptor kinase 5-like

## Data Availability

The transcriptomic data were submitted to NCBI and available (Accession: PRJNA894095).

## Supplementary Materials

The following supporting information can be downloaded at: https://www.mdpi.com/article/10.3390/biology11121716/s1, Figure S1: Bioinformatic analysis of DEGs; Figure S2: The chromosomal distribution of DEGS from testis transcriptomes; Figure S3: The distribution pattern of testis DEGs on Z chromosome; Figure S4: The DEG information of FoxO signaling pathway. Table S1: Primers used in this study; Table S2: Summary of sperm transcriptomes; Table S3: The chromosomal distribution of DEGs from sperm transcriptome; Table S4: The information of eight genes enriched in 14–15 M region of Z chromosomes; Table S5: The chromosomal distribution of DEGs from testis transcriptome.
