# Supplementary material for: Transcriptomic Analysis Revealed Candidate Genes Involved in Pseudomale Sperm Abnormalities in Chinese Tongue Sole (Cynoglossus semilaevis)
Source: Biology (Basel). 2022 Nov 26;11(12):1716. doi: 10.3390/biology11121716 (PMC9775080; doi:10.3390/biology11121716)
Supplement: Supplementary file 1 [file biology-11-01716-s001.zip › Supplementary Table S1 Primers used in this study.pdf]

Supplementary Table S1 Primers used in this study.

Gene ID	Primer	Sequence (5'-3')
103394973	Uncharacterized F	GTTTCTCTGAGGCAGCGAC
	Uncharacterized R	CGCTGCCATCCATCGCTTTA
112488693	brinp2 F	ACCTCCCTCCAGAAACGCTA
	brinp2 R	AACCAAAACCCAACAGACCT
103397964	nacc2 F	CGCTGCCATCCATCGCTTTA
	nacc2 R	TCGGTTGTCCAGGGGCTTTC
103397915	fmo5 F	TGCCACTCAGCGACTTCA
	fmo5 R	TTGTCCAATACGGCTCACC
103396267	lrrc7 F	GACACGCAGACGCTTCCCTT
	lrrc7 R	CGCCTTGAGTCCATTTCCTT
103397837	rtn4r F	GTCCAACCACGGGCTTTCA
	rtn4r R	TTTCGCTCGGCAGTCACA
103392878	syt3 F	GAGGCAACAGCCAGAGCAA
	syt3 R	CGATGACTGACAGCAAACTAACA
103380652	foxo1a F	GTCGTCCCCGTCTCCTATGAT
	foxo1a R	AAAACTGGGCTTGCTCTCTGG
103379355	foxo3a F	GAGGACGAGCCACCAAGAAAA
	foxo3a R	GCTGAGGGTGCTGGCATTAGA
103380652	foxo3b F	TTGCTTGTCAACATCAACCTG
	foxo3b R	TGAAGAGAAACCGTCGTGTG
103390850	foxo4 F	AGACGGTGCCTTACTTCAGAG
	foxo4 R	GGTGCTTTCCCAGTTTTGC
103387887	foxo6-like F	CAGTTATCATCCGCACCGTC
	foxo6-like R	GCCTTGTTCTCTTGAATCGTCT
103393304	actinF	TTCCAGCCTTCCTTCCTT
	actinR	TACCTCCAGACAGCACAG
