# Supplementary material for: Transcriptomic Analysis Revealed Candidate Genes Involved in Pseudomale Sperm Abnormalities in Chinese Tongue Sole (Cynoglossus semilaevis)
Source: Biology (Basel). 2022 Nov 26;11(12):1716. doi: 10.3390/biology11121716 (PMC9775080; doi:10.3390/biology11121716)
Supplement: Supplementary file 1 [file biology-11-01716-s001.zip › Supplementary Table S2 Summary of sperm transcriptomes.pdf]

Supplementary Table S2 Summary of sperm transcriptomes

Sample	Raw Reads	Raw Bases	Clean Reads	Clean Bases	Valid Bases	Q30
M1	45.97M	6.90G	45.20M	6.68G	96.95%	94.62%
M2	44.80M	6.72G	44.02M	6.52G	96.99%	94.50%
M3	43.63M	6.54G	42.87M	6.34G	96.83%	92.42%
M4	46.39M	6.96G	45.64M	6.76G	97.09%	94.71%
PM1	44.62M	6.69G	43.77M	6.46G	96.58%	94.02%
PM2	45.88M	6.88G	45.02M	6.65G	96.60%	94.22%
PM3	45.82M	6.87G	45.04M	6.65G	96.75%	94.07%
PM4	42.83M	6.43G	41.92M	6.19G	96.32%	93.80%
