# Supplementary material for: Transcriptomic Analysis Revealed Candidate Genes Involved in Pseudomale Sperm Abnormalities in Chinese Tongue Sole (Cynoglossus semilaevis)
Source: Biology (Basel). 2022 Nov 26;11(12):1716. doi: 10.3390/biology11121716 (PMC9775080; doi:10.3390/biology11121716)
Supplement: Supplementary file 1 [file biology-11-01716-s001.zip › Supplementary Table S4 The information of eight genes enriched in 14-15M region of Z chromosomes.pdf]

Supplementary Table S4 The information of eight genes enriched in 14-15M region of Z chromosomes

Gene ID	Annotation
LOC107990240	general transcription factor II-I repeat domain-containing protein 2-like
LOC103398026	SH2 domain-containing protein 3C
LOC103398119	Uncharacterized protein
LOC103398061	poly [ADP-ribose] polymerase 14-like
LOC103398033	G protein-coupled receptor kinase 5-like
LOC103398104	prkab1b, protein kinase, AMP-activated, beta 1 non-catalytic subunit, b
LOC103398005	poly [ADP-ribose] polymerase 14-like
LOC103398120	HPS4 biogenesis of lysosomal organelles complex 3 subunit 2
