# Supplementary figures and images for: Transcriptomic Analysis Revealed Candidate Genes Involved in Pseudomale Sperm Abnormalities in Chinese Tongue Sole (Cynoglossus semilaevis)
Source: Biology (Basel). 2022 Nov 26;11(12):1716. doi: 10.3390/biology11121716 (PMC9775080; doi:10.3390/biology11121716)

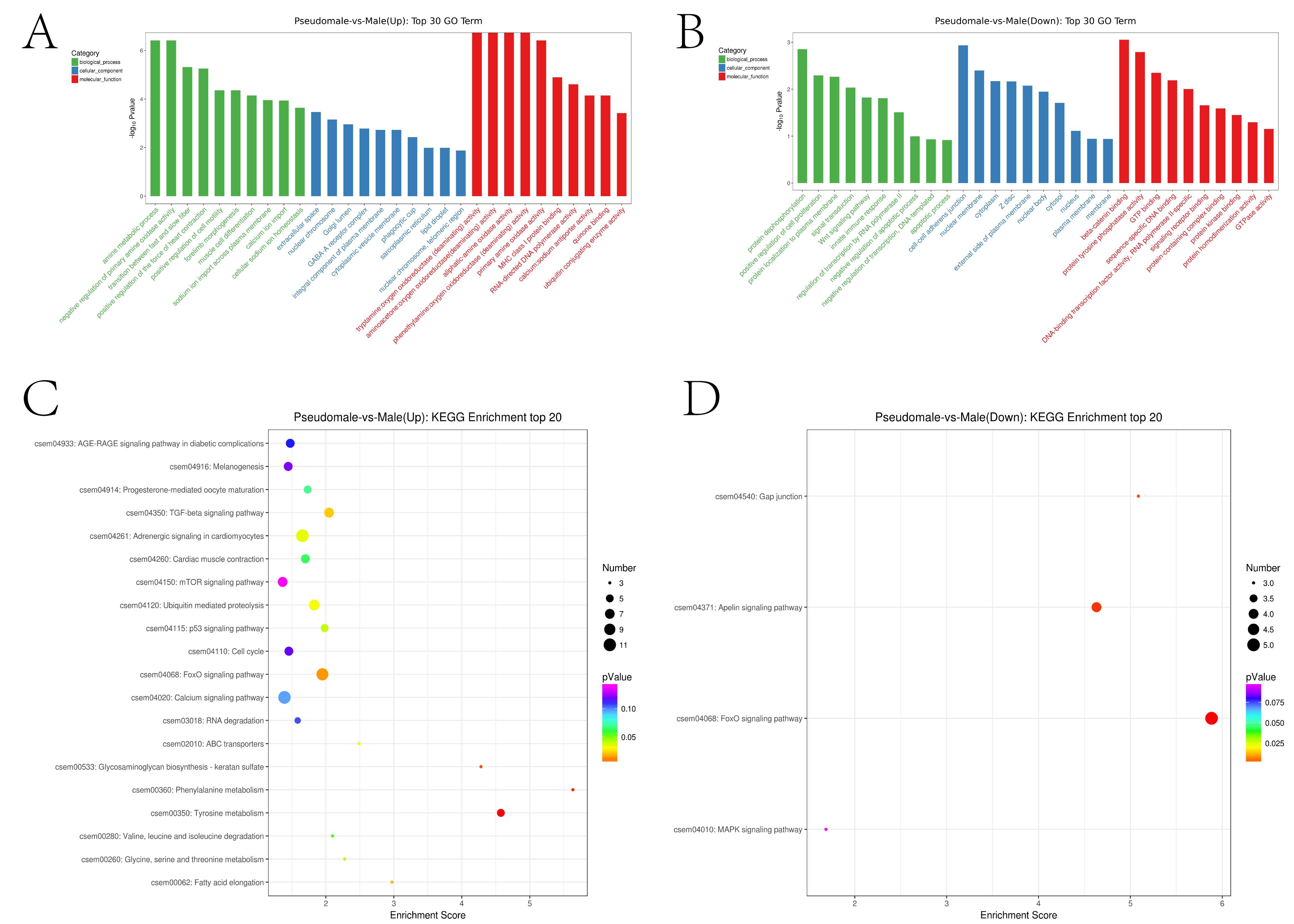

Supplement: Supplementary file 1 [file biology-11-01716-s001.zip › Supplementary Figure S1 Bioinformatic analysis of DEGs.tif]

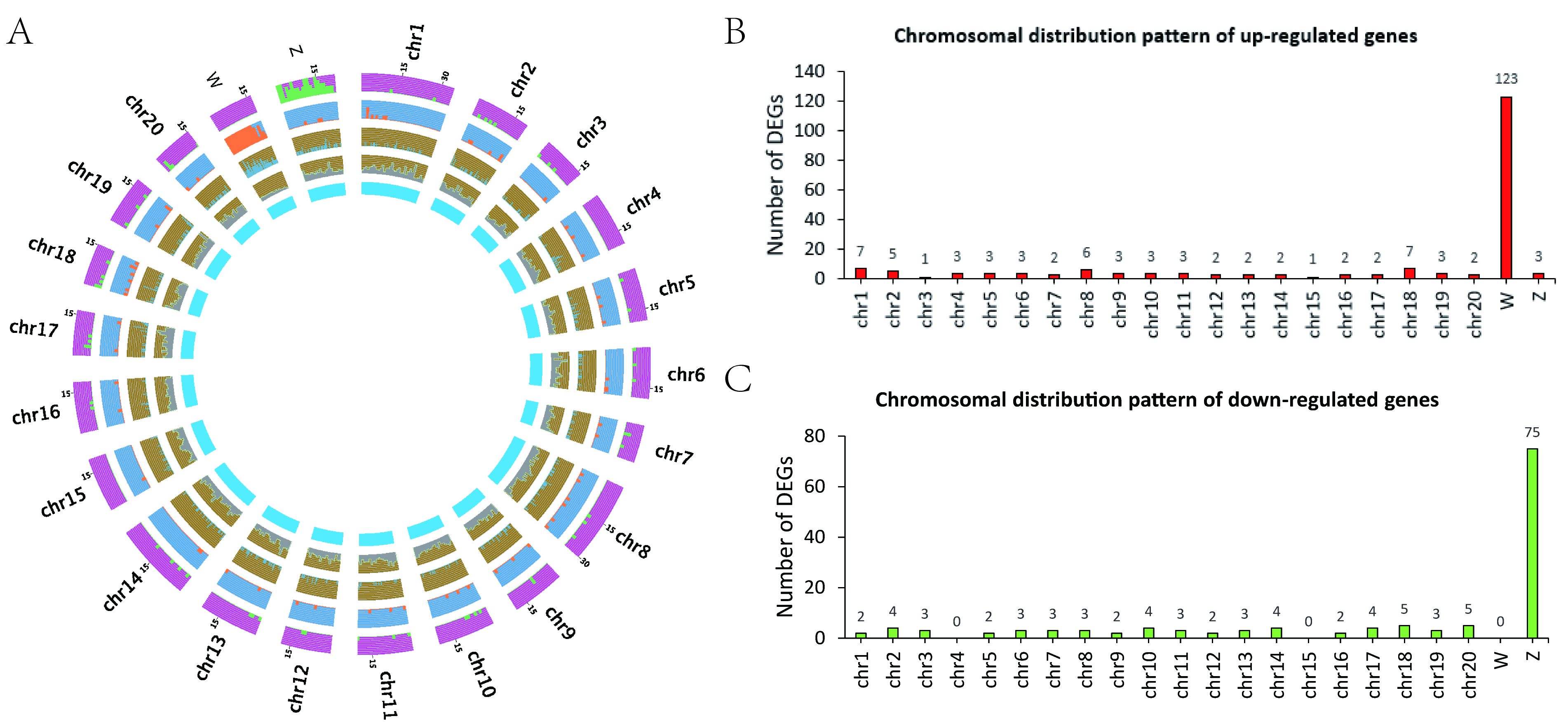

Supplement: Supplementary file 1 [file biology-11-01716-s001.zip › Supplementary Figure S2 The chromosomal distribution of DEGS from testis transcriptomes.tif]

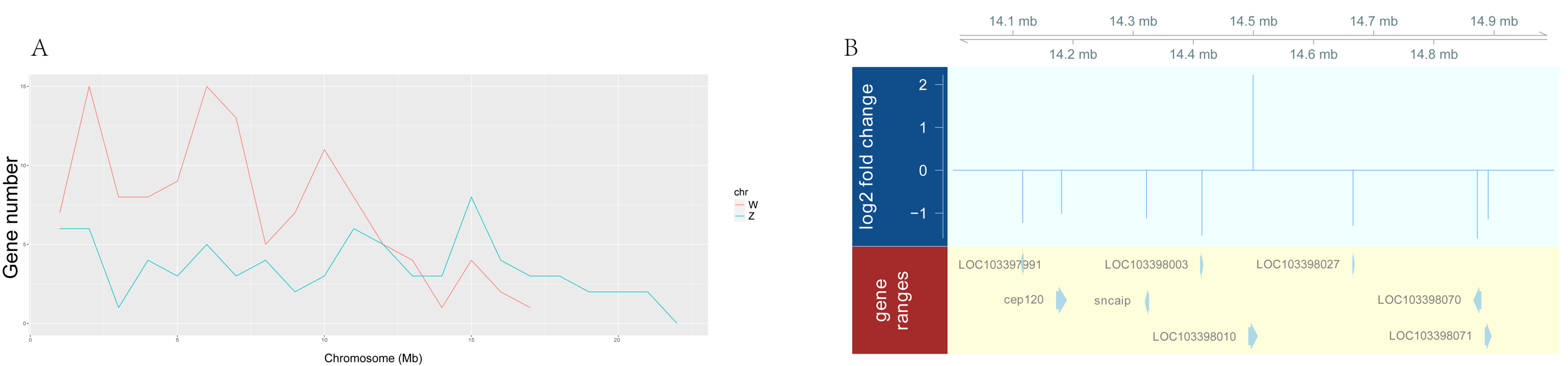

Supplement: Supplementary file 1 [file biology-11-01716-s001.zip › Supplementary Figure S3 The distribution pattern of testis DEGs on Z chromosome.tif]

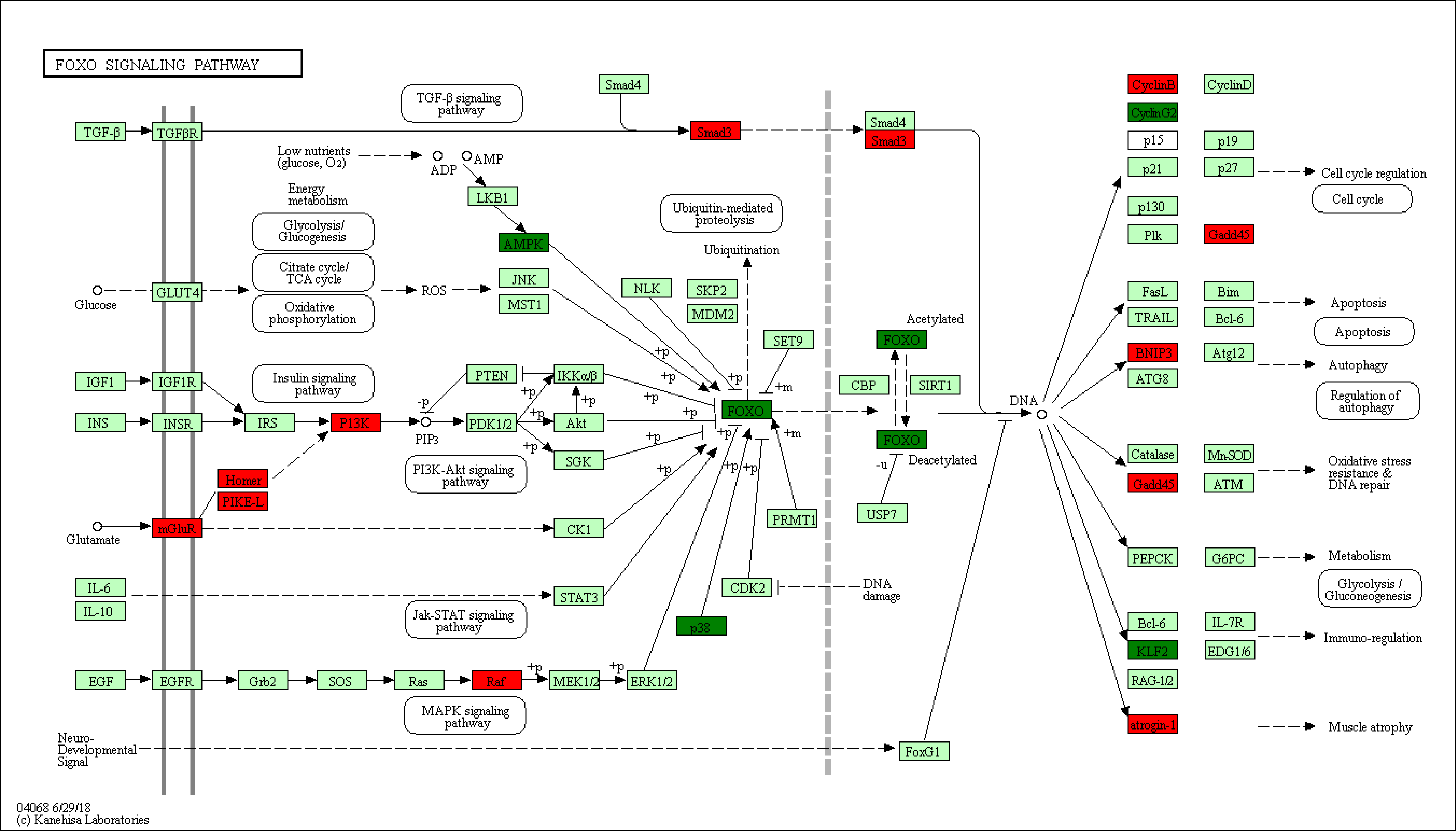

Supplement: Supplementary file 1 [file biology-11-01716-s001.zip › Supplementary Figure S4 The DEG information of FoxO signaling pathway.tif]
